# Contemporary biophysical approaches for studying 14-3-3 protein-protein interactions

**DOI:** 10.3389/fmolb.2022.1043673

**Published:** 2022-11-08

**Authors:** Bethany Thurairajah, Andrew J. Hudson, Richard G. Doveston

**Affiliations:** Leicester Institute for Structural and Chemical Biology and School of Chemistry, University of Leicester, Leicester, United Kingdom

**Keywords:** 14-3-3, fluorescence polarisation, FRET-fluorescence resonance energy transfer, isothermal titration calorimetery, surface plasmon resonace, molecular glues

## Abstract

14-3-3 proteins are a family of regulatory hubs that function through a vast network of protein-protein interactions. Their dysfunction or dysregulation is implicated in a wide range of diseases, and thus they are attractive drug targets, especially for molecular glues that promote protein-protein interactions for therapeutic intervention. However, an incomplete understanding of the molecular mechanisms that underpin 14-3-3 function hampers progress in drug design and development. Biophysical methodologies are an essential element of the 14-3-3 analytical toolbox, but in many cases have not been fully exploited. Here, we present a contemporary review of the predominant biophysical techniques used to study 14-3-3 protein-protein interactions, with a focus on examples that address key questions and challenges in the 14-3-3 field.

## 1 Introduction

14-3-3 proteins are a ubiquitous family of hub proteins that play vital roles in cellular homeostasis. They exert their effects by interacting with more than 1,200 protein binding partners, typically in a phosphorylation dependent manner (Sluchanko et al., 2019). Through these interactions 14-3-3 proteins regulate cellular localisation, post-translational modification, biomolecular interactions, and enzymatic or transcriptional activity of their partner proteins (Stevers et al., 2018a). As a result, this important protein family regulate the cellular lifecycle and multiple signalling pathways. 14-3-3 protein-protein interactions (PPI) are implicated in a range of disease areas including cancer (De Vries-van Leeuwen et al., 2013; Pennington et al., 2018; Falcicchio et al., 2020), neurodegeneration (Stevers et al., 2017; Andrei et al., 2018; Neves et al., 2021) and cystic fibrosis (Stevers et al., 2016; Stevers et al., 2022). Thus, they are an attractive target for drug development (Stevers et al., 2018a).

**FIGURE 1:**
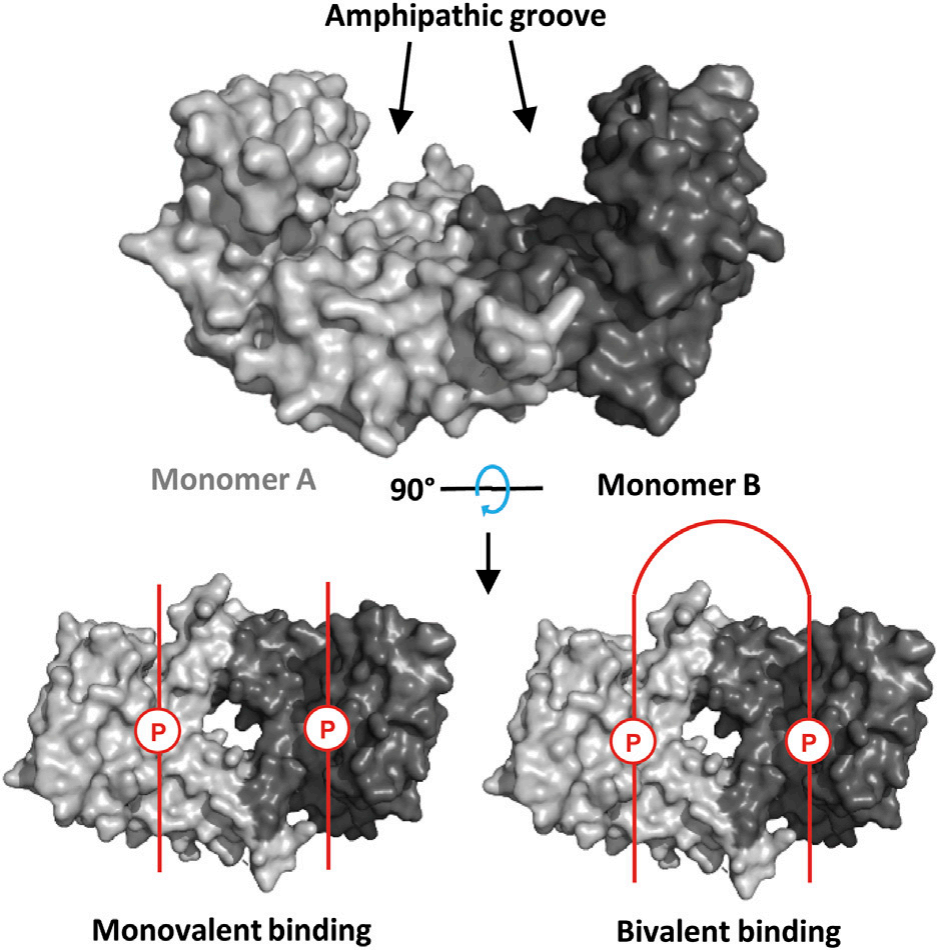
Top: Crystal structure of a 14-3-3σ dimer (PDB: 1YWT, partner peptide omitted). Bottom: Partner proteins (phosphorylated binding motifs represented in red; P = phosphosite) can bind to 14-3-3 in a monovalent fashion (i.e., 1:1 monomer-partner ratio, left), or a bivalent fashion (i.e., 1:0.5 monomer-partner ratio, right).

The human 14-3-3 family consists of seven isoforms (β, γ, ε, ζ, η, τ, σ) that exist as hetero- or homo-dimeric species (Sluchanko et al., 2019). Sequence homology across the family is high, especially in the conserved amphipathic binding groove that accommodates phosphorylated binding motifs of partner proteins (Figure 1) (Sluchanko and Donev, 2022). From a classical perspective, 14-3-3 binding motifs fall into 3 “modes”: mode I [RSX (pS/T)XP], mode II [RX (Y/F)X (pS/T)XP] and mode III [pS/TX-COOH] (Johnson et al., 2010). However, an increasing number of motifs that do not comply with these rules have been revealed, e.g. p53 (Kuusk et al., 2019) and MDMX (Srdanović et al., 2022). Furthermore, diphosphorylated peptides can span the 14-3-3 dimer to interact in a bivalent fashion (Figure 1), (Stevers et al., 2016; Stevers et al., 2018b; Srdanović et al., 2022) and interactions that occur around the less well-conserved periphery of 14-3-3 play an important role (Falcicchio et al., 2020).

The *in vitro* study of 14-3-3 PPIs has been based on a narrow range of structural and biophysical techniques. Structural biology has been used to study 14-3-3 PPIs with a focus on the binding of a phosphorylated partner peptide motif to a 14-3-3 dimer. This area has been dominated by protein crystallography which has to neglect the fact that 14-3-3 proteins are unlikely to be rigid entities in solution, but sample a range of dynamic conformations (Sluchanko et al., 2019). *In vitro* affinity studies using biophysical techniques allow the protein to sample its conformational space. However, some important considerations have not been investigated adequately, for example: the kinetics of partner-protein binding, the binding stoichiometry, 14-3-3 dimerisation and the basis of isoform specificity.

Addressing these fundamental questions regarding the molecular interactions of 14-3-3 proteins is an important endeavour because further understanding will expedite drug design and discovery. In particular, it would greatly improve our understanding of the cooperative effect that underpins the mechanism of action for molecular glues. Molecular glues, or PPI stabilisers/agonists, enhance the affinity of interacting proteins to induce a physiological effect. They are particularly relevant to 14-3-3 PPIs because they can exploit binding pockets at the interface of 14-3-3 and its partner protein (Stevers et al., 2018a). This requires molecular recognition of both protein partners and thus presents an opportunity to target specific 14-3-3 interactions in a selective manner. On the other hand, 14-3-3 PPI inhibitors are likely to affect numerous 14-3-3 interactions because they must disrupt interactions between common phosphorylated binding motifs and the conserved 14-3-3 phosphate binding pocket.

To highlight the contemporary challenges in this area, here we review both established and emerging biophysical approaches that have been used to study the important unanswered questions about 14-3-3 PPIs. We focus on highlighting recent applications of fluorescence polarisation (FP), fluorescence resonance energy transfer (FRET), isothermal titration calorimetry (ITC), and surface plasmon resonance (SPR).

## 2 Fluorescence polarisation

Fluorescence polarisation (FP) (or fluorescence anisotropy) is the most widely used biophysical technique for studying 14-3-3 PPIs. FP experiments measure the relative rate of tumbling of a small fluorescently labelled tracer molecule (i.e., < 2 kDa) in solution (Ciulli et al., 2013). When the free tracer is excited with polarised light, its fluorescent emission is depolarised, and detected in both parallel and perpendicular planes to the incident light. When the tracer molecule is bound to a much larger molecule such as a protein (>10 kDa), its rate of tumbling in solution is reduced and a higher degree of polarisation remains in the emitted light. Fluorescence polarisation (P, Eq. 1), and fluorescence anisotropy (r, Eq. 2) are closely related unit-less values quantifying the normalised difference between fluorescent emission intensity in the parallel and perpendicular planes relative to the plane of polarisation of the excitation: (Ciulli et al., 2013):

P=(F‖−F⊥)/(F‖+F⊥), (1)

r=(F‖−F⊥)/(F‖+2F⊥), (2)

Synthetic peptides that mimic the binding motifs of the phosphorylated partner protein to 14-3-3 have a sufficiently small size relative to 14-3-3 and can be modified to incorporate a fluorescent label. Their use in place of the natural protein partner makes FP a particularly versatile technique for studying 14-3-3 PPIs. In this review of recent 14-3-3 literature, we have identified 15 studies that used FP as part of the analytical workflow (Soini et al., 2021a; Cossar et al., 2021; Falcicchio et al., 2021; Gogl et al., 2021; Guillory et al., 2021; Horvath et al., 2021; Ruks et al., 2021; Sijbesma et al., 2021; Centorrino et al., 2022; Hazegh Nikroo et al., 2022; Joshi et al., 2022; Srdanovic et al., 2022; Srdanović et al., 2022; Stevers et al., 2022). Because it is such a well-established technique, here we focus on select examples that demonstrate how FP can be used to address three key challenges in current 14-3-3 research:

(i) Quantifying the cooperative nature of 14-3-3 molecular glues;
(ii) Understanding 14-3-3 isoform specificity and partner protein binding mechanisms;
(iii) Studying the kinetic characteristics of 14-3-3 PPIs.

### 2.1 Using FP to quantify the cooperative nature of 14-3-3 molecular glues

**FIGURE 2:**
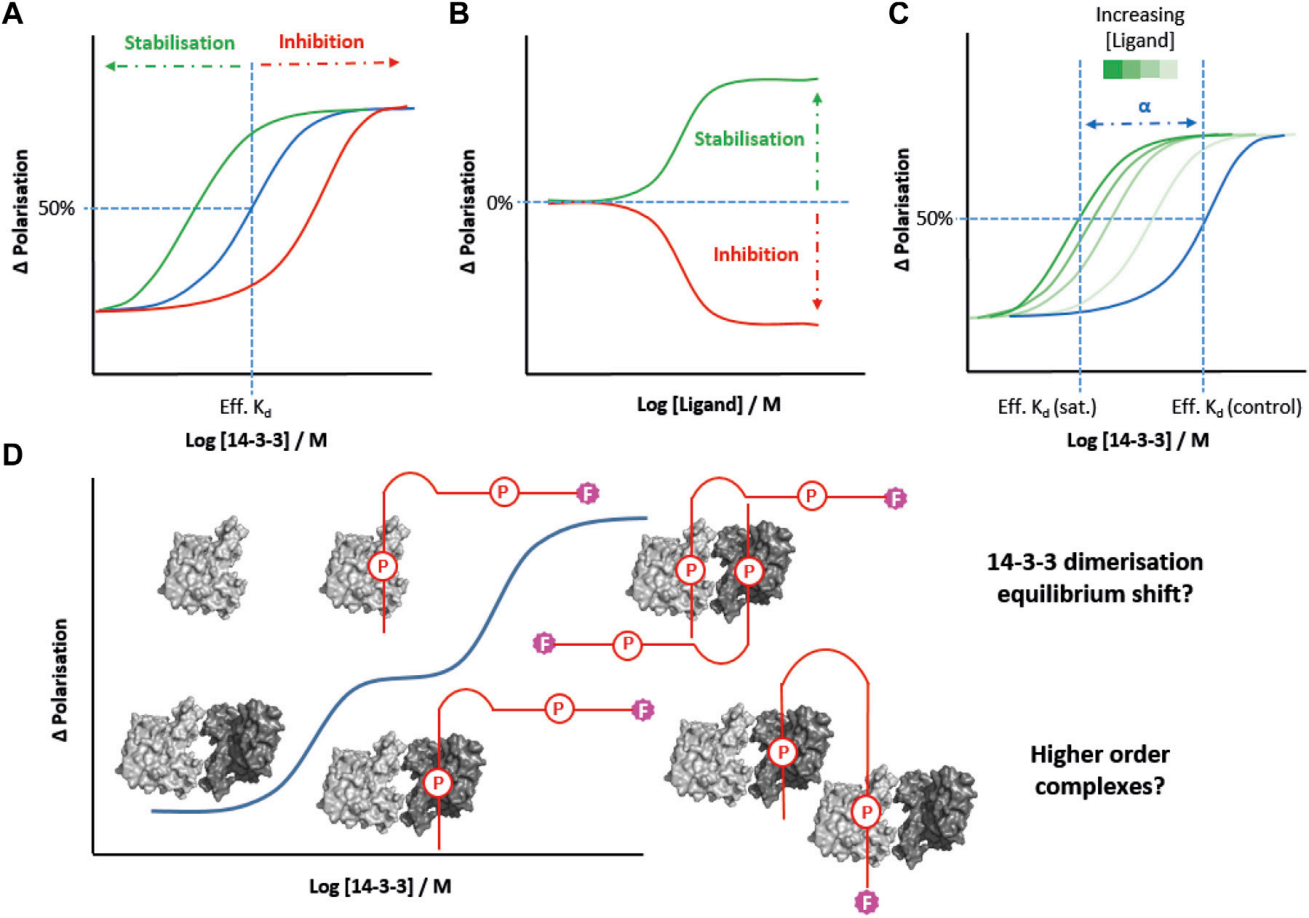
FP strategies for studying 14-3-3 PPIs. **(A)** 14-3-3 protein titrations into fixed concentrations of a molecular glue (green) and a PPI inhibitor (red) allow for comparison of effective K_d_s (Eff. K_d_) compared to a control experiment (blue). **(B)** Titration of molecular glues or PPI inhibitors into fixed concentrations of 14-3-3 protein can determine EC_50_ values. **(C)** 2D titration of 14-3-3 protein in the presence of increasing fixed concentrations of molecular glue allow the cooperativity factor (*α*) to be determined. **(D)** Hypotheses to explain the biphasic FP behaviour of MDMX peptides (red) binding to 14-3-3. P = phosphosite.

Molecular glues offer great promise as drugs for targeting 14-3-3 PPIs in disease (Stevers et al., 2018a). FP has been widely used to screen and profile molecular glues from established examples such as fusicoccin A through to novel fragments, macrocycles and peptide-conjugates. Typically, the effect of a glue is quantified either: 1) by performing 14-3-3 protein titrations in the presence of a fixed concentration of the glue or inhibitor and partner peptide, and comparing the effective K_d_ observed to that of a control experiment (see in Figure 2A); or 2) by performing ligand titrations to fixed concentrations of 14-3-3 protein and partner peptide to calculate “EC_50_” values (see in Figure 2B) (Soini et al., 2021a; Falcicchio et al., 2021; Guillory et al., 2021; Sijbesma et al., 2021; Wolter et al., 2021; Centorrino et al., 2022; Srdanovic et al., 2022; Stevers et al., 2022). However, both of these approaches are concentration dependent and the values obtained differ depending on experimental design (i.e., the concentration of the fluorophore in protein titrations, or the concentration of the 14-3-3 protein in ligand titrations). Quantifying the overall increase in the stability of a protein-protein complex in a concentration independent manner provides a more accurate assessment of the effectiveness of molecular glues. Recently, the cooperativity factor (*α*) has emerged as a better quantitative measure of PPI in the presence of a glue (de Vink et al., 2019). In simple terms, *α* is calculated by performing FP protein titrations at increasing but fixed concentrations of molecular glue until a saturation point is reached beyond which higher concentrations of glue do not further reduce the effective K_d_ for the protein-peptide interaction. It is the ratio of this interval point value relative to the control K_d_ (in the absence of a glue) that determines the value of *α* (Figure 2C). (Cossar et al., 2021; de Vink et al., 2019) This 2-dimensional FP approach has been used to give much clearer insight into cooperativity and the structure-activity relationships associated with 14-3-3 molecular glues. Most recently it has been applied effectively in the development of covalent lysine-reactive stabilisers of the 14-3-3γ–Pin1 PPI (Cossar et al., 2021), and semi-synthetic fusicoccane-derived stabilisers of the 14-3-3γ–p65 PPI (Wolter et al., 2020).

### 2.2 Using FP to understand 14-3-3 isoform specificity and partner protein binding mechanisms

FP is also an important tool for addressing key questions regarding isoform-specific behaviour and our understanding of non-classical partner-protein binding mechanisms. For example, FP was the underpinning technique used to profile the binding affinities of E6 human papilloma virus oncoprotein peptide motifs to all seven human 14-3-3 isoforms (Gogl et al., 2021). A similar study was undertaken to analyse binding of the 14-3-3 isoforms to peptides derived from the binding motifs of MDMX and MDM2 (Srdanović et al., 2022), and for SLP76 (Soini et al., 2021a). These comprehensive comparative studies further confirm the established hierarchy of 14-3-3 isoform binding affinities (γ/η > ζ > τ > β > ε > σ) observed for most known partner proteins. Understanding the structural and functional basis of this hierarchy is a significant challenge in the field. FP will no doubt be an important tool for advancing understanding of 14-3-3 isoform recognition beyond the classical phosphorylation-depended binding model. Indeed, the insight FP can provide has recently been demonstrated in the case of the unusual binding mechanism of 14-3-3 with MDMX and MDM2 (Srdanović et al., 2022). In this case, 14-3-3 titration experiments produced unusual biphasic binding curves for diphosphorylated peptides mimicking the 14-3-3 binding motif of MDMX and MDM2, suggesting two iterative binding events (Figure 2D). It was postulated that this could be due to concentration dependence of 14-3-3 dimerisation, i.e. the extent of dimerisation increases as the concentration of 14-3-3 protein increases (see Figure 2D) (Srdanović et al., 2022). An alternative explanation is that the peptide bridges two 14-3-3 dimers at high protein concentrations. This could occur *via* the second phosphosite, or a non-specific interaction. The biphasic effect is 14-3-3 isoform dependent, and is most pronounced for those isoforms that tend to heterodimerise, which supports the first argument. However, it has not previously been reported with other 14-3-3 binding partners which is suggestive something specific to this type of binding motif.

Although undoubtedly an essential tool, FP does have limitations. Firstly, it requires a small peptidic binding partner motif as the tracer (as opposed to a full length protein), and secondly, it is associated with a large number of false-positive hits in large scale library screens. However, it does enable high-throughput analysis using relatively small amounts of material. In the future, by using time-resolved FP, it could be possible to learn more about the mechanism of 14-3-3 PPIs. This potential was recently demonstrated through an elegant synthetic 14-3-3 protein scaffold that incorporated a kinase-activated auto-inhibition motif. (Hazegh Nikroo et al., 2022). Over the period of a few hours, FP was used to monitor the displacement of a tracer molecule during the course of phosphorylation of the 14-3-3 scaffold (and, hence, as auto-inhibition increased). It is possible that FP kinetic data collected on much shorter timescales could shed more new light on the dynamics of 14-3-3 PPIs and molecular glues. In relation to this, experiments that measure fluorescence anisotropy decay on the nanosecond timescale can also provide fascinating insight into the environment of the fluorophore under different conditions (Horvath et al., 2021; Joshi et al., 2022).

## 3 Fluorescence resonance energy transfer

Fluorescence resonance energy transfer (FRET) is a useful solution-based method to study 14-3-3 PPIs, but one that has been under-exploited (Roy et al., 2008). FRET involves the excitation of a donor molecule, followed by electronic-energy transfer from the donor to an acceptor; where the emission band of the donor must overlap with the absorption band of the acceptor. The transfer of energy between the two molecules is non-radiative, and dependent on their spatial separation. The efficiency of FRET is 50% at the Föster distance, which is, typically, between 10 and 100 Å; where a shorter Föster distance is obtained at higher values of the overlap integral between the donor emission and acceptor absorption bands (Gordon et al., 1998; R. M. B. T.-L. T and Clegg, 2009). Time-resolved FRET (TR-FRET) involves using a donor with a long fluorescent lifetime, eliminating any interferences from short-lived fluorescence emanating from impurities or autofluorescence. Lanthanide chelates are common fluorophores for TR-FRET that are very stable and have a large stokes shift; the time delay prior to measurement removes background fluorescence, hence TR-FRET has an improved signal-to-noise ratio. FRET approaches have been used for characterising 14-3-3 interactions with partner proteins, and also for monitoring 14-3-3 dimerisation and intramolecular conformational changes. Despite the potential this technique offers, there are only a very limited number of examples. Here we review all the reported FRET-based approaches for studying 14-3-3 proteins.

### 3.1 Using FRET to study 14-3-3 PPIs and their modulators

**FIGURE 3:**
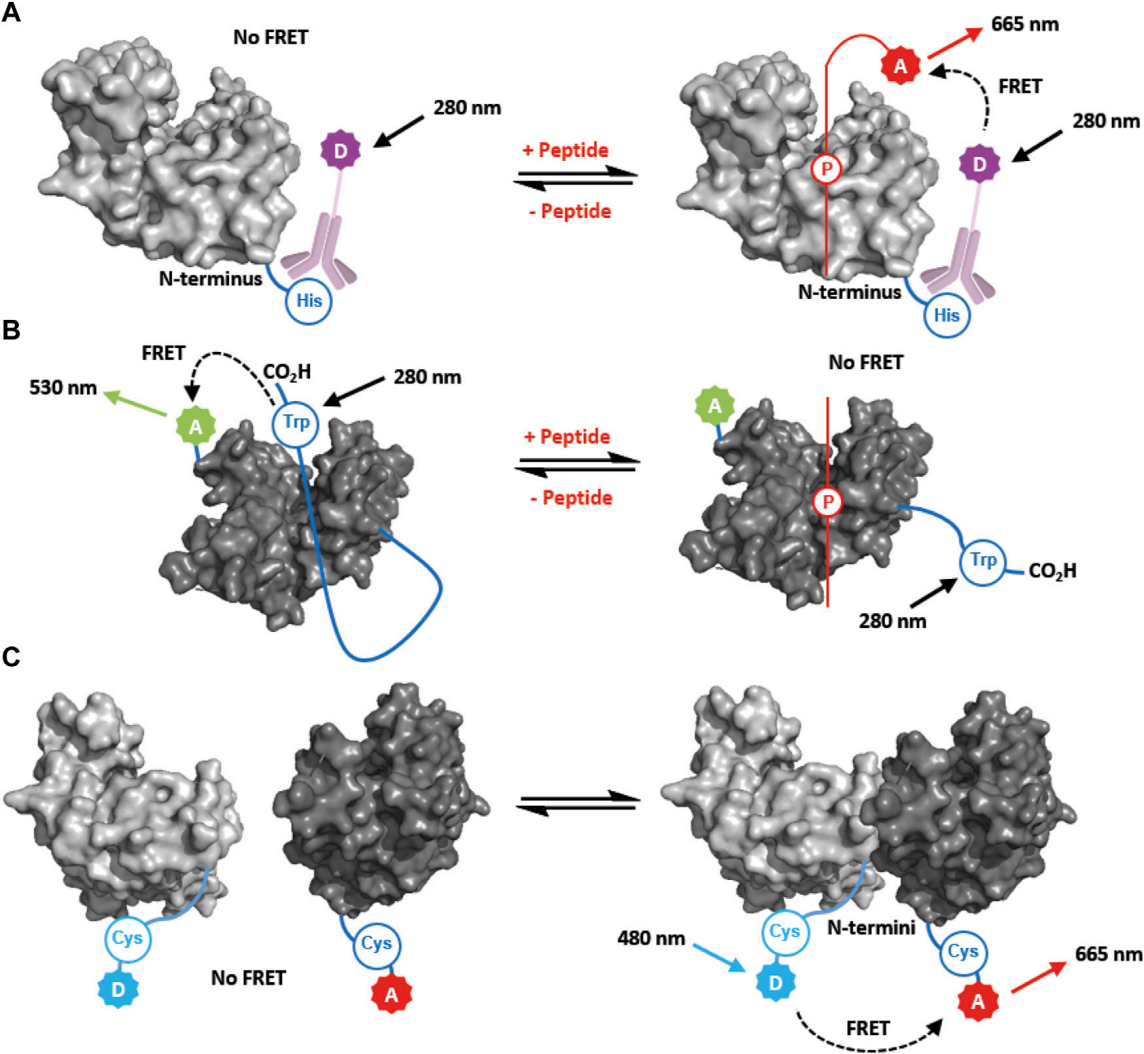
FRET-based approaches for studying 14-3-3 PPIs and structural dynamics. **(A)** FRET used to measure partner peptide binding. The partner peptide (red) is labelled with a FRET acceptor, A. 14-3-3 is labelled with a FRET donor, D, *via* an anti-His antibody that binds to the N-terminal His-tag. **(B)** FRET used to measure 14-3-3 C-terminal dynamics. A tryptophan residue (Trp) at the C-terminus is used as a FRET donor. A Cys residue proximal to the 14-3-3 binding groove is labelled with a FRET acceptor, A In the absence of a partner peptide the C-terminus of 14-3-3 occupies the groove, and FRET occurs. FRET is lost upon binding of a parner peptide (red) which displaces the C-terminal tail. **(C)** FRET used to monitor 14-3-3 dimerisation. 14-3-3 monomers are differentially labelled with FRET donor, D, or acceptor, A, *via* the N-terminus. FRET occurs upon dimerisation when one 14-3-3 construct is titrated into the other.

One of the earliest examples of a FRET study was a time-resolved assay for the 14-3-3ζ—Bad PPI (Du et al., 2013). A europium-conjugated anti-His antibody was used to label 14-3-3ζ, while a 17mer phosphopeptide mimicking the Bad protein was labelled with Dy647 at the N-terminus (Figure 3A). This provided an effective and robust FRET system for monitoring the affinity of the Bad peptide for all seven human 14-3-3 isoforms (K_d_ = 1.7–6.0 nM), without the need for covalent protein modification (Du et al., 2013). The assay was validated by screening known inhibitors, and ultimately miniaturised to 1536-well format for high-throughput screening and the discovery of novel inhibitors of the 14-3-3ζ—Bad PPI. The TR-FRET data obtained was compared with fluorescence polarisation (FP) data. It was concluded that TR-FRET provided a more reproducible assay, had less propensity to yield false-positive hits, and consumed less protein and peptide reagent (Du et al., 2013). Therefore, it was deemed a superior technique for identifying hit inhibitor compounds.

More recently TR-FRET has been used to study the interaction between 14-3-3γ and a full length phosphorylated protein partner, SLP76 (Soini et al., 2021b). This was achieved by covalently labelling 14-3-3γ with a terbium cryptate donor molecule bearing an amine-reactive isothiocyanate group. The extent of 14-3-3γ labelling was not reported, however it is reasonable to assume that multiple lysine residues and the N-terminus of 14-3-3γ reacted with the label. The 20 kDa phosphorylated SLP76 construct was labelled using an AlexaFluor 647 (AF647) dye bearing a succinimidyl ester (Soini et al., 2021b). Again, the extent of protein labelling was not reported, and it is unlikely the approach can be used for quantitative measurements of FRET or fluorescence lifetime. However, this approach does provide a robust qualitative assay for screening for modulators of the PPI, and in particular molecular glues. A library of 20,000 molecules was screened giving rise to 16 novel molecular glues to take forward into further optimization rounds (Soini et al., 2021b).

FRET has also been used to monitor the stoichiometric ratio of partner peptides binding to a 14-3-3τ. ITC revealed that two diphosphorylated peptides mimicking the 14-3-3 binding motifs of IRSp53 bound to 14-3-3 with a molar ratio of 1:0.5 (14-3-3: IRSp53) suggesting that one peptide binds to a 14-3-3 dimer (see section 4.2 for discussion of the ITC data) (Kast and Dominguez, 2019). To confirm this, a FRET donor-labelled monophosphorylated peptide (mimicking a single phosphorylation site) was complexed with 14-3-3 at half-saturation (i.e., 1:0.5 stoichiometry). When a complementary acceptor-labelled monophosphorylated peptide was titrated into this complex, an increase in FRET occurred providing evidence that the second peptide was occupying the free binding groove on the vacant 14-3-3 monomer (Kast and Dominguez, 2019). In contrast, when the same acceptor labelled peptide was titrated to a complex of 14-3-3 and donor-labelled diphosphorylated peptide at the same molar ratio, no FRET was observed. This provided strong evidence that a free binding site was no longer available, and that the monophosphorylated peptide could not compete with the higher affinity diphosphorylated binder (Kast and Dominguez, 2019). This FRET data corroborated the ITC data suggesting the diphosphorylated peptides bound in a bivalent fashion to a 14-3-3 dimer.

### 3.2 Using FRET to study 14-3-3 protein tertiary and quaternary structure

The wealth of structural data on 14-3-3 proteins provides a comprehensive model for how 14-3-3 proteins fold into their familiar tertiary structure, and form dimeric complexes. However, it is often neglected that 14-3-3 proteins are dynamic structures—in particular, movement of the C-terminal tail is not resolved in crystal and NMR structures. 14-3-3 dimerisation is also a dynamic process that plays an important functional role. FRET-based approaches have been used to study both these important facets of 14-3-3 dynamics.

As early as 2004, Silhan et al. (2004) reported FRET and fluorescence lifetime approaches to study the dynamic nature of the C-terminus of 14-3-3ζ, and show how it competes with phospho-peptides for binding in the amphipathic groove. To achieve this a 14-3-3ζ mutant construct was prepared that contained a single intrinsically-fluorescent Trp242 residue located at the end of the C-terminal tail as the FRET donor. The 14-3-3ζ construct was also engineered to contain a single solvent-exposed cysteine residue at Cys25 that was covalently modified with a dansyl group (AEDANS) to make a FRET acceptor (Figure 3B) (Silhan et al., 2004). In the presence of a phosphopeptide mimicking the binding motif of pRaf, the FRET efficiency of the system was diminished, thus indicating displacement of the C-terminal tail away from the main body of the 14-3-3ζ protein (Silhan et al., 2004).

The fluorescence lifetime of a donor fluorophore is also directly related to the FRET efficiency to acceptor fluorophore. Hence, to complement the study above, the fluorescence lifetime of Trp242 was measured, and used to detect the distance between Cys25 and Trp242. The data indicated that in the absence of the pRaf peptide the C-terminal tail must be within 30 Å of the Cys25, and thus occupy the amphipathic groove of 14-3-3ζ, or other proximal space (Silhan et al., 2004). When the pRaf peptide was introduced this distance increased to 36 Å which provided further evidence for displacement of the C-terminal tail (Silhan et al., 2004). Fluorescence lifetime measurements are more reliable than FRET intensity measurements because they are not effected by saturation, inner filtering or fluorophore photobleaching. It is also a more quantitative approach to biophysical analysis and thus can provide additional information compared to direct FRET intensity measurements in binding assays.

Dimerisation is key to the overall cellular function of 14-3-3, however the dynamic nature of the association of monomers has not been widely investigated. Recently, Trošanová et al. (2022) developed a FRET-based approach to study 14-3-3ζ dimerisation. To achieve this, 14-3-3ζ was engineered to include an N-terminal sequence containing a single cysteine residue. This was selectively modified with fluorescent donor (AF488) or acceptor (AF647) maleimides, which by virtue of the linker did not interfere with the dimerisation interface. As one 14-3-3ζ species was titrated to another, re-equilibration of the monomers and dimers occurred. As corresponding donor and acceptor monomers formed dimers, the FRET intensity increased (Figure 3C). By fitting the data to a binding model, a K_d_ of 4.6 nM was determined for the dimeric complex (Trošanová et al., 2022). This value was corroborated in a subsequent kinetic experiment whereby the time course for equilibration was monitored following the addition of unlabeled 14-3-3ζ. The data obtained suggest that 14-3-3ζ dimers have a slow off-rate (2.8 × 10–3 s^−1^) (Trošanová et al., 2022). A K_d_ of 3.6 nM was calculated which was in close alignment to that obtained in the initial equilibrium experiments. Intriguingly, the introduction of a phosphopeptide binding partner led to increased dimer stability. An analogous approach was also used to study how mutation of 14-3-3ζ Ser58 to Glu affected dimerisation. (Kozeleková et al., 2022). The effect of shifting 14-3-3 dimerisation equilibria (when 14-3-3 protein is titrated from very low to very high concentrations) has been proposed as an explanation for some unexpected biphasic FP binding curves (see Section 2.2) (Srdanović et al., 2022).

These examples highlight that FRET-based approaches can be used to study the affinity of 14-3-3 PPIs, investigate stoichiometry of protein complexes, screen for both small molecule inhibitors and stabilisers, and study the structural dynamics of 14-3-3 proteins. FRET has three distinct advantages over the more commonly used FP approach:

(1) Both labelled peptide mimics and full length protein partners can be used in the assay because there is no requirement that one partner is significantly smaller than the other;
(2) The technique is much more sensitive than fluorescence polarisation, and time-resolved approaches can eliminate short-lived background fluorescence that often gives rise to a large number of false-positives;
(3) Typically this approach will consume less peptide and protein reagent.

A drawback of the technique is that both binding partners require labelling with fluorescent probes. Furthermore, the choice of fluorophore and its positioning require careful optimisation for any given 14-3-3 PPI. These challenges might explain why FP is more frequently used for studying 14-3-3 PPIs.

## 4 Isothermal titration calorimetry

Isothermal titration calorimetry (ITC) is a powerful quantitative technique for characterising biomolecular interactions, and has been widely used to study 14-3-3 PPIs. It measures the heat change during complex formation and thus provides important insight into the thermodynamic parameters that govern the affinity between two interacting molecules, namely changes in enthalpy (ΔH) and entropy (ΔS) (Leavitt and Freire, 2001). These both contribute to the overall Gibbs free energy change of a given system which is governed by: ΔG = ΔH–TΔS. For an interaction to be spontaneous, ΔG must be negative. Therefore, high affinity interactions (i.e., those with a low dissociation constant, K_d_) require a large and negative ΔH, and/or a large and positive ΔS. For associative processes, the relationship between ΔH and ΔS is one of compensation, whereby a favourable change in enthalpy is necessary to compensate for an unfavourable change in entropy (or *vice versa*) (Chodera and Mobley, 2013). ITC also provides information about the stoichiometry of any given system. In this review of recent 14-3-3 research, we have identified seven studies that include ITC as an element of the analytical workflow (Kast and Dominguez, 2019; Soini et al., 2021a; Chen et al., 2021; Falcicchio et al., 2021; Guillory et al., 2021; Ruks et al., 2021; Srdanović et al., 2022). Here, we focus on select examples, and refer to other relevant data, that highlight the importance of ITC in addressing two connected challenges in the field: 1) understanding the thermodynamic drivers behind 14-3-3 interactions which can be molecular glues; and 2) determining 14-3-3 binding stoichiometry.

### 4.1 Using ITC to study the driving forces behind 14-3-3 PPIs

**FIGURE 4:**
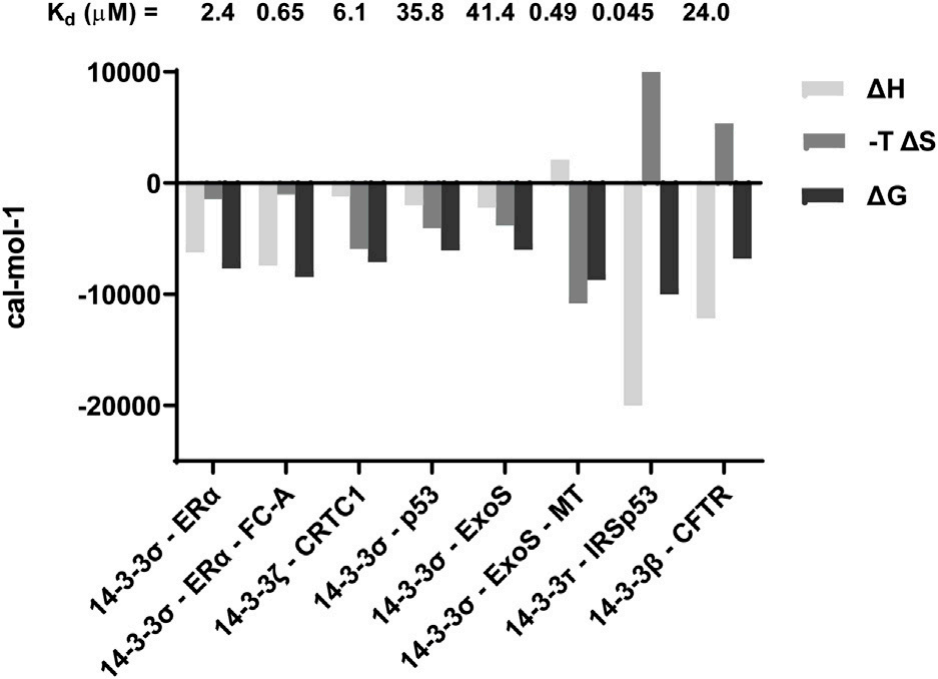
Selected ITC data highlighting contributions from ΔH and ΔS to ΔG, and thus the K_d_. Previously reported data for the following PPIs is included: 14-3-3—ERα (Falcicchio et al., 2021); 14-3-3ζ—CRTC1 (Chen et al., 2021); 14-3-3σ—p53 (Kuusk et al., 2019); 14-3-3σ—ExoS (Guillory et al., 2021); 14-3-3τ—IRSp53 (Kast and Dominguez, 2019); 14-3-3β—CFTR (Stevers et al., 2016).

Based on emerging data, monovalent PPIs can be broadly categorised into those where binding affinity (i.e., ΔG) is dominated by ΔH, and those largely governed by ΔS. Classical “mode 3” binding motifs tend to be enthalpy dominated, for example the interaction between 14-3-3σ and ERα (Figure 3). (Falcicchio et al., 2021). This suggests an exothermic (i.e., energetically favourable) process where the formation of new non-covalent interactions such as hydrogen bonds is critical. Stabilisation of these interactions, such as stabilisation of the 14-3-3—ERα interaction by fusicoccin A (FC-A), tends to be as a result of an increased enthalpic contribution to ΔG (Figure 4) (Falcicchio et al., 2021).

Beyond the classical 14-3-3 binding motifs, entropy often dominates the binding landscape. An interesting example is binding of a peptide mimicking region 2 of the CREB-regulated transcriptional coactivator 1 (CRTC1) to 14-3-3ζ (Chen et al., 2021). Here, the high affinity observed is due to the absence of an entropic penalty upon binding (Figure 4).

The contribution from ΔH is small because the peptide can adopt a pseudo-secondary structure in solution. Therefore, very little conformational rearrangement of the peptide takes place upon binding, an event usually associated with favourable ΔH. The small ΔH contribution is supplemented by a positive ΔS arising from rearrangement of the solvent network (i.e., a hydrophobic effect). So, although an associative process like this would normally be associated with a high entropic penalty, this is off-set by a large hydrophobic effect that leads to a favourable entropy change. When mutations to the peptide were made that disrupted the proposed secondary structure, affinity was not lost, but the interactions became ΔH-driven (Chen et al., 2021). Thus, pre-organization of peptide secondary structure is an important factor because it dictates an entropy dominated “lock and key” mechanism, as opposed to conformational rearrangement and selection (Du et al., 2016). Similar observations were made with peptides mimicking the p53 motif that interacts with 14-3-3σ (Kuusk et al., 2019).

Another example of a ΔS-driven 14-3-3 interaction is that with a phosphopeptide representing ExoS conjugated to a molecular tweezer (MT) (Guillory et al., 2021). The affinity of the ExoS peptide alone for 14-3-3σ was governed by comparable contributions from ΔH and ΔS. Conjugation of the MT changed this profile completely, rendering the process endothermic (Figure 4). The interaction remained spontaneous because of very large entropic contribution that was so significant the relative affinity increased by > 80-fold (Guillory et al., 2021). This appears to suggest that 14-3-3 PPIs are not always driven solely by the formation of non-covalent interactions arising from the phosphate motif binding in the amphipathic groove. They can also be driven by an increase in entropy arising from disruption of the solvent network, and/or an increase in conformational flexibility of 14-3-3 or the binding partner.

### 4.2 Using ITC to determine 14-3-3 binding stoichiometry

Differentiating mono-vs. bivalent 14-3-3 interactions using biophysical techniques is challenging. ITC is a useful tool because it provides stoichiometric information about the interactions taking place. The example of IRSp53 binding to 14-3-3τ is a good illustration of this (Kast and Dominguez, 2019). Monophoshproylated peptides mimicking putative 14-3-3τ binding motifs both returned a molar ratio (*N* value) of ∼1 as would be expected if one peptide bound to one 14-3-3τ monomer. In both cases, the affinity was governed by favourable contributions from both changes in enthalpy and entropy. When a diphosphorylated peptide was investigated, the *N* value was 0.5, which is consistent with a single peptide binding to a 14-3-3 dimer. The entropic contribution to binding of the diphosphorylated peptide was unfavourable, and thus the process was dominated by ΔH (Kast and Dominguez, 2019). This example is comparable to that of the 14-3-3β interaction with CFTR, where a diphosphorylated peptide bound with a molar ratio of 0.74 in a manner characterised by an unfavourable entropic contribution compensated by a large negative change in ΔH (Stevers et al., 2016). These observations can be explained by the formation of energetically favourable non-covalent interactions with two phosphate groups (rather than just one), and also a much more significant reduction in the conformational freedom of the bound peptide compared to mono-phosphorylated examples (hence a negative ΔS). In contrast to these examples, ITC analysis of a diphosphorylated peptide representing MDMX binding to 14-3-3η did not share this thermodynamic profile, and the molar ratio was found to be close to 1 (Srdanović et al., 2022). However, both phosphorylation sites were required for high affinity binding, which may point to an alternative model of stochastic rebinding being a driver for 14-3-3 PPIs (Srdanović et al., 2022).

Whilst ITC is no doubt an important and powerful technique for analysing 14-3-3 PPIs, it does come with some drawbacks. For example the quantity of protein required has limited studies to peptidic binding partners only; and, to obtain accurate data, their purity need to be very accurately determined. The experimental approach usually requires titration of high concentrations of peptide partner into a buffered solution of 14-3-3 protein often at concentrations of over 100 µM. In addition to the practical drawback in terms of the amount of material required, such conditions may not be physiologically relevant or may mask subtle differences in stoichiometry or one vs. two site binding. The sensitivity of the technique is also a limitation when measuring interactions giving rise to very little heat change, i.e., interactions dominated by ΔS with a contribution from ΔH that is close to zero.

## 5 Surface plasmon resonance

Surface plasmon resonance (SPR) is an optical microfluidic technique that measures the change in refractive index of a medium close to a surface in real time. It is a particularly useful technique for studying PPIs, including 14-3-3 PPIs, because it provides kinetic information as well as binding affinities. The approach involves immobilisation of one binding partner onto a surface (sensor chip), while the other binding partner is present in a laminar flow over the surface. A beam of light is reflected towards this surface at a critical angle and thus generates a surface plasmon; the critical angle is dependent on the refractive index of the medium. Binding of the two partners causes a change in the refractive index which reflects the extent of binding that has occurred (Boozer et al., 2006; Douzi et al., 2017). SPR has been often used as a tool to analyse 14-3-3 PPIs and their modulators. However, the majority of studies use the technique in a superficial way to corroborate K_d_ values obtained *via* other methods, typically using maximum response measurements taken across a range of analyte concentrations. Here we focus on the recent studies that consider the quantitative kinetic information provided by this technique and draw comparisons to historical examples. However, first the methods used to immobilise one binding partner onto the sensor chip are discussed because this has a significant bearing on experimental performance.

### 5.1 Methods of immobilisation for SPR

In the majority of cases, 14-3-3 protein is immobilised onto the sensor chip. This is commonly achieved using a Ni-NTA coated chip that binds to the His6 tag at the N-terminus of the 14-3-3 protein (Soini et al., 2021a; Chen et al., 2021; Srdanović et al., 2022), or *via* stochastic amide coupling to an amine-functionalised chip mediated using 1-ethyl-3-(3-dimethylaminopropyl)carbodiimide (EDC) and N-hydroxysuccinaminde (NHS) (Kuusk et al., 2019; Hartman et al., 2020). In one example, the 14-3-3 protein was biotinylated and immobilised onto a streptavidin-coated sensor chip (Neves et al., 2021). These approaches are robust, however they require careful optimisation to achieve reproducible and appropriate immobilisation density across the surface. The alternative strategy is to immobilise a phosphopeptide binding partner on to the surface *via* biotin-avidin chemistry (Fuglsang et al., 1999; Chen et al., 2021) or amide coupling (NHS/EDC) (Rose et al., 2010). While this approach has been successfully applied it should be noted that in the case of immobilised CRTC peptides, significantly slower on (k_on_) and off rates (k_off_) were observed for 14-3-3 binding compared to the analogous experiment using immobilised 14-3-3 (*via* its His-tag) (Chen et al., 2021). This might be explained by reduced accessibility of the phosphorylated binding motif when immobilised close to the surface, compared to a much more accessible 14-3-3 binding groove when the protein is immobilised *via* its distal His-tag (Chen et al., 2021). Therefore, the design of SPR experiments can have a significant influence on the data obtained.

### 5.2 14-3-3 binding kinetics

Examples where the kinetic parameters of 14-3-3 PPIs have been quantitatively analysed by SPR are rare, despite the unique insight this provides. In a recent example, 14-3-3η was immobilised *via* an N-terminal His-tag and the kinetic profiles were measured for the interaction with peptides mimicking the unusual interaction with MDMX (Srdanović et al., 2022). In this example, the 22-fold higher affinity of a diphosphorylated peptide, compared to a monophosphorylated derivative, was found to be mostly a consequence of a 25-fold decrease in the off rate (k_off_ = 0.0048 s^−1^ relative to k_off_ = 0.1208 s^−1^) (Table 1) (Srdanović et al., 2022). The contribution to the increased affinity from k_on_ was much smaller in comparison, with only a 4-fold increase. This indicates that increased residency time of the peptide drives affinity, thus supporting the proposed model of “statistical rebinding”.

**TABLE 1:** Examples of quantitative SPR analysis of 14-3-3 PPIs.

Ligand	14-3-3 isoform	Immobilisation	K_d_ (nM)	K_D_ (nM)	k_on_ (M^−1^s^−1^)	k_off_ (s^−1^)
MDMX (pS367) Srdanović et al. (2022)	η	14-3-3 *via* His-tag	189	310	3.89 × 10^5^	0.1208
MDMX (pS342, PS367) Srdanović et al. (2022)			8.6	3.17	1.51 × 10^5^	0.0048
Synaptopodin (mode 2) Hartman et al. (2020)	ζ	14-3-3 *via* EDC/NHS	1,380	1,380	3.92 × 10^4^	0.0542
A1H3 Hartman et al. (2020)			n.d	16,000	2.6 × 10^3^	4.1 × 10^−2^
A2H3 Hartman et al. (2020)				15,000	5.8 × 10^2^	8.7 × 10^−3^
AHA2 (mode 3) Fuglsang et al. (1999)	n.d	AHA2 peptide *via* biotin	n.d	88.0	1.6 × 10^5^	1.4 × 10^−2^
AHA2 +FC-A Fuglsang et al. (1999)				7.0	1.3 × 10^5^	9.5 × 10^−4^

nd = not disclosed.

SPR has also been used to study the kinetic profile of 14-3-3 binding to the mode 3 binding motif of the plant ATPase AHA2 (Fuglsang et al., 1999). The 15-fold higher affinity of the mode 3 motif for 14-3-3is driven by equal contributions from a 4-fold increase in k_on_ and 4-fold decrease in k_off_. When the molecular glue FC-A was added to the mode 3 AHA2 system, the affinity of 14-3-3 for the peptide increased by 12-fold, almost entirely due to a much slower k_off_ (Fuglsang et al., 1999). This suggests that PPI stabilisation efficacy is driven by the stability and thus half-life of the ternary complex formed. Surprisingly, to the best of our knowledge, this is the only example where the effect of a molecular glue on the kinetic profile of a 14-3-3 PPI has been reported in a quantitative manner.

14-3-3ζ binding to a phosphopeptide mimicking the classical mode 2 binding motif of synaptopodin has also been profiled, alongside ligands developed as part of a dynamic combinatorial chemistry programme targeting this PPI (Table 1) (Hartman et al., 2020). The effect of ligands (A1H3 and A2H3, Table 1) on the affinity of the 14-3-3 PPI was not reported, but their affinity for 14-3-3 protein alone was studied. Intriguingly, despite having almost identical affinities, A1H3 and A2H3 showed very different kinetic profiles in terms of k_on_ and k_off_, perhaps suggesting different binding mechanisms. Furthermore, SPR response unit data was used to show that the novel ligands did not compete for the same binding site as the peptide, and thus occupied an allosteric space such as that commonly occupied by FC-A (Hartman et al., 2020).

SPR is clearly a powerful method for studying 14-3-3 PPIs, and in particular the kinetic profile of these interactions, and how this is affected by molecular glues. It is a label free technique which means that the biomolecular components of the assay do not require non-native modification. It is also versatile in that it can be used to elucidate competitive vs. non-competitive binding (Hartman et al., 2020), and it has even been used to confirm interaction stoichiometry (Soini et al., 2021a). Assay optimisation and reproducibility, especially with respect to immobilisation, do present a major challenge to experimentalists. However, it is anticipated that SPR, and analogous techniques such as biolayer interferometry, will become ever more important tools for gaining a full understanding of 14-3-3 PPIs and how they can be stabilised.

## 6 Conclusion

14-3-3 PPIs play critical roles in cellular homeostasis and disease. Understanding the precise molecular mechanisms that underpin these interactions is essential for expediting drug discovery efforts, and uncovering the true extent of the 14-3-3 “interactome”. In this review, we have summarised emerging biophysical approaches that have been used to address key research questions in the field that limit our current understanding. Specifically, we have focused on investigations that explore the cooperative effect of molecular glues, the basis of isoform specificity, interaction stoichiometry, 14-3-3 dimerisation, and interaction kinetics. In isolation, each of the techniques FP, FRET, ITC, and SPR act as a unique lens through which to study 14-3-3 PPIs, but these methods alone do not provide a full picture. Other approaches not discussed here (e.g., microscale thermophoresis (Centorrino et al., 2022; Kozeleková et al., 2022), differential scanning fluorimetry (Cossar et al., 2021; Waløen et al., 2021; Joshi et al., 2022), analytical ultracentrifugation (Horvath et al., 2021; Leysen et al., 2021; Neves et al., 2021; Pohl et al., 2021; Srdanović et al., 2022), single angle x-ray scattering (Kast and Dominguez, 2019; Leysen et al., 2021) also contribute new understanding and support hypotheses. NMR (Kuusk et al., 2019; Neves et al., 2021) and native mass spectrometry (Bellamy-Carter et al., 2021) are also important techniques that shows great promise for studying 14-3-3 PPIs. Ultimately, a full understanding of 14-3-3 function will only be achieved by taking an interdisciplinary approach whereby high quality biophysical data complements unexplored single molecule approaches, together with structural and cellular biology studies.
